# Supplementary material for: Educational Apps and Dog Behavioural Problem Prevention: Associations Between the Zigzag Dog-Training App and Behavioural Problems
Source: Animals (Basel). 2025 Feb 12;15(4):520. doi: 10.3390/ani15040520 (PMC11851379; doi:10.3390/ani15040520)
Supplement: Supplementary file 1 [file animals-15-00520-s001.zip › animals-3398467-supplementary File S1.pdf]

Full copy of survey

Puppy Development Survey

Start of Block: Default Question Block

Investigating the impact of online training on puppy behaviour and dog owner relationships

We are inviting you to take part in a research study. Before you decide, it is important that you know why we are doing the study and what is involved. Please read the following information carefully.

What is the purpose of the study?
Although we know that experiences that puppies have early in life can influence their later behaviour, we still need to learn a great deal about how we can best help puppies to grow into well adjusted adult dogs. A new online training programme has been developed and we would like to see whether it is benefitting puppies and their owners and if so in what way. For this reason we are asking people who have participated in the online programme as well as those who have not, to complete a short, anonymous online survey so that we can start to understand the benefits of this specific training intervention.

Am I eligible to take part?
You are being invited to take part because you are over 18 years of age and the owner of a dog between 3 months of age and 2 years of age

Do I have to take part?
Participation is completely voluntary. You should only take part if you want to and choosing not to take part will not disadvantage you in anyway.

What will I be asked to do?
After you have read this information sheet you will be asked to provide informed consent by electronically agreeing to a number of statements. You will then have access to the survey questions which should take no more than 15-20 minutes to complete. The questions cover a number of areas including some general information about you and your dog, as well as questions relating to how you feel about your dog (using the MONASH Dog Owner Relationship Scale), your dog's behaviour and any training you may have done with your dog. No personal information which can identify you will be asked and as the questionnaire is anonymous, once you have submitted it we will not be able to remove your information as it will not be identifiable. You are free to stop answering questions at any point.

Will I be paid expenses for taking part?
You will not be paid to participate in the study.

What are the possible benefits / risks of taking part?
There will be no specific direct benefits to you from taking part in the survey. However, the information that we obtain from the survey is likely to help us to help dogs and owners in the future. There should be no risk to you from taking part in the survey. If by taking part in the survey you realise that you would like to seek advice for your dog's behaviour or training we would recommend that you look for an appropriately certificated trainer or behaviourist.

Will anyone know I have taken part?
No one will know you have taken part, as your responses are anonymous.

Where will my data be stored?
The data obtained from the study will be stored securely on the university OneDrive with password protected access. Only the researchers will have access to it. Paper copies will be stored in a secure cabinet/office at the University. The data from this study may be put in an Open Access repository for other researchers to use in future research. If this happens, all data will be anonymous.

What will happen if I don't want to carry on with the study?
As you have completed the study anonymously it will not be possible to remove the data provided, as we will not be able to identify you in any way.

What will happen to the results of the research study? The results of the survey will be shared with the funding body who is running the online training (Zigzag) who may choose to distribute the information via their website. Any data that the research team deems of value to the field of animal behaviour will be written up for publication in peer reviewed journals and presented at scientific meetings.

Who is organising and funding the research?
This research is being organised by Daniel Mills and Helen Zulch at the University of Lincoln and is being funded by Zigzag as a subsidiary of PetCare Services LTD.

Who has reviewed the study?
All research conducted by the University of Lincoln is looked at by an independent group of people, called a Research Ethics Committee, to protect your interests. This study has been reviewed and given favourable opinion by a University of Lincoln Research Ethics Committee [2023_9472].

What if there is a problem?
It is very unlikely that this study would cause you any harm. If you have a concern or a complaint about any aspect of this study, you should ask to speak to the researchers who will do their best to answer your questions. The researchers contact details are given at the end of this information sheet. If you remain unhappy and wish to complain formally, you can make a formal complaint through the University complaints procedure or by contacting ethics@lincoln.ac.uk.

Further information and contact details
Daniel Mills dmills@lincoln.ac.uk ; 01522 83 5356
Helen Zulch hzulch@lincoln.ac.uk; 01522 83 5469

CONSENT TO PARTICIPATE IN RESEARCH Please **SELECT ALL** of the following in order to continue to consent to participate and proceed to the rest of the survey.

- ☐ I confirm that I have read the information sheet dated 06/01/22 (version 1) for the above study. I have had the opportunity to consider the information, ask questions and have had these answered satisfactorily. (1)
- ☐ I understand that as I have completed the study anonymously it will not be possible to remove any information I have provided, as you will not be able to identify me in any way. (2)
- ☐ I understand that individuals from the University of Lincoln may look at research data collected during the study, to ensure that the study is conducted appropriately. (3)
- ☐ I understand that the information collected about me may be used to support other research in the future, and may be shared anonymously with other researchers. (4)
- ☐ I agree to take part in the above study. (5)

Skip To: End of Survey If Investigating the impact of online training on puppy behaviour and dog owner relationships We are... != I confirm that I have read the information sheet dated 06/01/22

(version 1) for the above study. I have had the opportunity to consider the information, ask questions and have had these answered satisfactorily.

Skip To: End of Survey If Investigating the impact of online training on puppy behaviour and dog owner relationships We are... != I understand that as I have completed the study anonymously it will not be possible to remove any information I have provided, as you will not be able to identify me in any way.

Skip To: End of Survey If Investigating the impact of online training on puppy behaviour and dog owner relationships We are... != I understand that individuals from the University of Lincoln may look at research data collected during the study, to ensure that the study is conducted appropriately.

Skip To: End of Survey If Investigating the impact of online training on puppy behaviour and dog owner relationships We are... != I understand that the information collected about me may be used to support other research in the future, and may be shared anonymously with other researchers.

Skip To: End of Survey If Investigating the impact of online training on puppy behaviour and dog owner relationships We are... != I agree to take part in the above study.

Page Break

Thank you for agreeing to take part in our survey. This is a collaboration between Zigzag and the University of Lincoln, aimed at helping us understand important impacts on puppy development. We are not gathering personally identifiable information about you, but we would like to know a little bit about your dog, what training you have done and his/her behaviour.

If you have more then one puppy please answer the questions in relation to only one puppy and the same one throughout.

Before that we will start we need to check a few things.
Are you over 18 years old and the primary carer of the puppy you will be answering about?

○ Yes (1)

○ No (2)

Skip To: End of Survey If Thank you for agreeing to take part in our survey. This is a collaboration between Zigzag and the... = No

Page Break

How old is your puppy now? (please give to the closest month)
Months (1)

▼ 1 (1) ... 24+ (25)

Skip To: End of Survey If How old is your puppy now? (please give to the closest month) = 24+
Skip To: End of Survey If How old is your puppy now? (please give to the closest month) = 1
Skip To: End of Survey If How old is your puppy now? (please give to the closest month) = 2

Page Break

A few questions about you:
Please indicate into which of the age groups you fit:

- 18-25 (1)
- 26-35 (2)
- 36-45 (3)
- 46-55 (4)
- 56-65 (5)
- 66+ (6)

Please tell us with which gender you identify:

- Male (1)
- Female (2)
- Non-binary / third gender (3)
- Prefer not to say (4)

Within which country do you currently live?

- United Kingdom (1)
- Australia (2)
- New Zealand (3)
- South Africa (4)
- USA (5)
- Other (6)

Have you attended any of the following with your puppy? You can select as many as you wish to.

▢ In person puppy party / parties, i.e. one off events often run by vet practices in which puppies mix and some general advice is given (1)

▢ In person puppy training classes, i.e a series of formal classes following a specific curriculum in which puppies are trained to show certain behaviours (2)

▢ Other formal in person training, (if yes please describe below) (3)
____

▢ Other informal in person socialisation activities (if yes please describe below, e.g. going to the park to socialise) (4) ____

▢ Remote training activities EXCLUDING Zigzag Puppy Education that was a free online e.g. YouTube (if yes please describe below) (5)
____

▢ Remote training online acitivities EXCLUDING Zigzag Puppy Education that was for a paid online course e.g. Absolute Dogs (if yes please describe below) (6)
____

▢ ⊗No training at all (7)

Have you enrolled with the Zigzag Puppy Education App?

○ No (1)

○ Yes (2)

Skip To: Q10 If Have you enrolled with the Zigzag Puppy Education App? = No

---

Which of the following chapters in the Zigzag Puppy Education App have you accessed and used? (tick all that apply). Please note that because there are two versions of the App you may either be answering "Chapter 1-10" or "PupStarter/PupMaster Parts 1-4/5"

	Done some exercises but not all (1)	Completed all exercises (2)	Not read (3)
Chapter 1/PupStarter Part 1 (1)	○	○	○
Chapter 2/PupStarter Part 2 (2)	○	○	○
Chapter 3/PupStarter Part 3 (3)	○	○	○
Chapter 4/PupStarter Part 4 (4)	○	○	○
Chapter 5/PupMaster Part 1 (5)	○	○	○
Chapter 6/PupMaster Part 2 (6)	○	○	○
Chapter 7/PupMaster Part 3 (7)	○	○	○
Chapter 8/PupMaster Part 4 (8)	○	○	○
Chapter 9, 10 & 11/PupMaster Part 5 (9)	○	○	○

Did you manage to **complete <u>all</u> of the first 4 chapters** of the App or alternatively, depending on your Zigzag version, the PupStarter course *<u>before your puppy was 12 weeks old?</u>*

○ Yes (1)

○ No (2)

○ Not sure (3)

Display This Question:

If Did you manage to complete all of the first 4 chapters of the App or alternatively, depending on... = No

Or Did you manage to complete all of the first 4 chapters of the App or alternatively, depending on... = Not sure

Did you manage to complete <u>all of the first 4 chapters</u> of the App or alternatively, depending on your Zigzag version, the PupStarter course before your puppy was **16** weeks old?

- Yes (1)
- No (2)
- Not sure (3)

Page Break

Name of puppy

____

How old was your puppy when they were adopted/purchased?

- ○ Less than 6 weeks (1)
- ○ 6-8 weeks (2)
- ○ 9-12 weeks (3)
- ○ 13-16 weeks (4)
- ○ Over 16 weeks (5)

What is your puppy's gender and neuter status?

- ○ Male entire (1)
- ○ Female entire (2)
- ○ Male neutered (3)
- ○ Female neutered (4)

Skip To: Q70 If What is your puppy's gender and neuter status? = Male entire

Skip To: Q70 If What is your puppy's gender and neuter status? = Female entire

If neutered, please indicate at what month they were neutered (to the closest month)
Month (1)

▼ 1 (1) ... 24 (24)

Page Break

Which of the following best describes the breed type of dog you own?
Breed (1)

▼ AFFENPINSCHER (1) ... OTHER (266)

Page Break

Where did you get your puppy from?

- ◯ Family/friend (1)
- ◯ Private breeder (2)
- ◯ Shelter/Rescue centre (3)
- ◯ Bred myself (4)
- ◯ Found abandoned (5)
- ◯ Other (6)

Have you ever owned a dog before?

- ◯ No (1)
- ◯ Yes (2)

Does your puppy have any ongoing health issues you are aware of?

- ◯ No (1)
- ◯ Yes, please specify (2) ____

How long is your puppy left alone (without human company) on the average day?

- never (1)
- 1-2 hours (2)
- 2-4 hours (3)
- 4-6 hours (4)
- 6-8 hours (5)
- 8-10 hours (6)
- 10-12 hours (7)
- more than 12 hours (8)

End of Block: Default Question Block

Start of Block: Block 2

Select the picture above that best describes the closeness of your relationship with your dog: “Self” represents you and “dog” represents your dog."

- A (1)
- B (2)
- C (3)
- D (4)
- E (5)
- F (6)
- G (7)

The following questions refer to how you feel about your puppy.
Please rate the following statements to the best of your ability concerning the extent to which you agree or disagree with them.

	Strongly agree (1)	Agree (2)	Neither agree nor disagree (3)	Disagree (4)	Strongly disagree (5)
If everyone else left me, my dog would still be there for me (1)	○	○	○	○	○
My dog helps me get through tough times (2)	○	○	○	○	○
My dog is there whenever I need to be comforted (3)	○	○	○	○	○
It bothers me that my dog stops me doing things I enjoyed doing before I owned it (4)	○	○	○	○	○
It is annoying that I sometimes have to change my plans because of my dog (5)	○	○	○	○	○
My dog costs too much money (6)	○	○	○	○	○
My dog provides me with constant companionship (7)	○	○	○	○	○
I would like to have my dog near me all the time (8)	○	○	○	○	○
My dog gives me a reason to get up in the morning (9)	○	○	○	○	○
I wish my dog and I never had to be apart (10)	○	○	○	○	○

My dog is constantly attentive to me (11)	○	○	○	○	○
My dog makes too much mess (12)	○	○	○	○	○
There are major aspects of owning a dog I don't like (13)	○	○	○	○	○

Page Break

Please complete the following list of questions to the best of your ability concerning how often the following applies to you.

	Once a day (1)	Once a week (2)	Once a month (3)	Once a year (4)	Never (5)
How often do you feel that having a dog is more trouble than it is worth? (1)	o	o	o	o	o
How often do you play games with your dog? (2)	o	o	o	o	o
How often do you tell your dog things you don't tell anyone else? (3)	o	o	o	o	o
How often do you feel that looking after your dog is a chore? (4)	o	o	o	o	o
How often does your dog stop you doing things you want to do? (5)	o	o	o	o	o

Page Break

Please rate the following statement:

	Very traumatic (1)	Traumatic (2)	Neither traumatic nor untraumatic (3)	Untraumatic (4)	Very untraumatic (5)
How traumatic do you think it will be for you when your dog dies? (1)	○	○	○	○	○

Please indicate how frequently the following apply to you, with regard to this puppy.

	Once a week (1)	Once a fortnight (2)	Once a month (3)	A couple times a year (4)	Never (5)
How often do you take your dog to visit people? (1)	○	○	○	○	○
How often do you buy your dog presents? (2)	○	○	○	○	○
How often do you give your dog food treats? (3)	○	○	○	○	○

Page Break

Please complete the following questions to the best of your ability.

	At least once a day (1)	Once every few days (2)	Once a week (3)	Once a month (4)	Never (5)
How often do you kiss your dog? (1)	o	o	o	o	o
How often do you take your dog in the car? (2)	o	o	o	o	o
How often do you hug your dog? (3)	o	o	o	o	o
How often do you have your dog with you while relaxing, i.e., watching TV? (4)	o	o	o	o	o
How often do you groom your dog? (5)	o	o	o	o	o

Please select an answer for the following question

	Very hard (1)	Hard (2)	Neither hard nor easy (3)	Easy (4)	Very easy (5)
How hard is it to look after your dog? (1)	o	o	o	o	o

Page Break

Does your puppy have an issue with chewing?

- No problem at all (behaviour is absent or not worthy of note) (1)
- Mild (causes disruption but can be lived with) (2)
- Medium (difficult and persistent problem but you are learning to live with it/work around it) (3)
- Severe (persistent problem causing disruption to your normal life and your expectations) (4)

Does your puppy have an issue with play biting?

- No problem at all (behaviour is absent or not worthy of note) (1)
- Mild (causes disruption but can be lived with) (2)
- Medium (difficult and persistent problem but you are learning to live with it/work around it) (3)
- Severe (persistent problem causing disruption to your normal life and your expectations) (4)

Does your puppy have an issue with house soiling?

- No problem at all (behaviour is absent or not worthy of note) (1)
- Mild (causes disruption but can be lived with) (2)
- Medium (difficult and persistent problem but you are learning to live with it/work around it) (3)
- Severe (persistent problem causing disruption to your normal life and your expectations) (4)

Does your puppy have an issue with aggression (growling, snarling, snapping or biting outside of play) towards human family members, or close friends?

- No problem at all (behaviour is absent or not worthy of note) (1)
- Mild (causes disruption but can be lived with) (2)
- Medium (difficult and persistent problem but you are learning to live with it/work around it) (3)
- Severe (persistent problem causing disruption to your normal life and your expectations) (4)

Skip To: Q45 If Does your puppy have an issue with aggression (growling, snarling, snapping or biting outside of... = No problem at all (behaviour is absent or not worthy of note)

Page Break

Please indicate which of the following signs have been shown? (please tick all that apply)

- ▢ Biting (making contact) (1)
- ▢ Air snap/lunging (2)
- ▢ Growl/snarl (3)
- ▢ Other, please specify (4) ____

Please indicate to whom aggression has been shown (tick all that apply)

- ▢ Adult male (1)
- ▢ Adult female (2)
- ▢ Male child (3)
- ▢ Female child (4)

Page Break

Does your puppy have an issue with aggression (growling, snarling, snapping or biting outside of play)towards strangers?

- No problem at all (behaviour is absent or not worthy of note) (1)
- Mild (causes disruption but can be lived with) (2)
- Medium (difficult and persistent problem but you are learning to live with it/work around it) (3)
- Severe (persistent problem causing disruption to your normal life and your expectations) (4)

Skip To: Q44 If Does your puppy have an issue with aggression (growling, snarling, snapping or biting outside of... = No problem at all (behaviour is absent or not worthy of note)

Please indicate which of the following signs have been shown? (please tick all that apply)

- Biting (making contact) (1)
- Air snap/lunging (2)
- Growl/snarl (3)
- Other, please specify (4) ____

Page Break

Does your puppy have an issue with aggression (growling, snarling, snapping or biting outside of play) to other dogs?

- ○ No problem at all (behaviour is absent or not worthy of note) (1)
- ○ Mild (causes disruption but can be lived with) (2)
- ○ Medium (difficult and persistent problem but you are learning to live with it/work around it) (3)
- ○ Severe (persistent problem causing disruption to your normal life and your expectations) (4)

Skip To: Q43 If Does your puppy have an issue with aggression (growling, snarling, snapping or biting outside of... = No problem at all (behaviour is absent or not worthy of note)

Please indicate which of the following signs have been shown? (please tick all that apply)

- ☐ Biting (making contact) (1)
- ☐ Air snap/lunging (2)
- ☐ Growl/snarl (3)
- ☐ Other, please specify (4) ____

Page Break

Does your puppy have an issue with jumping up?

- No problem at all (behaviour is absent or not worthy of note) (1)
- Mild (causes disruption but can be lived with) (2)
- Medium (difficult and persistent problem but you are learning to live with it/work around it) (3)
- Severe (persistent problem causing disruption to your normal life and your expectations) (4)

Does your puppy have an issue with car travel?

- No problem at all (behaviour is absent or not worthy of note) (1)
- Mild (causes disruption but can be lived with) (2)
- Medium (difficult and persistent problem but you are learning to live with it/work around it) (3)
- Severe (persistent problem causing disruption to your normal life and your expectations) (4)

Does your puppy have an issue with chasing others or moving objects?

- No problem at all (behaviour is absent or not worthy of note) (1)
- Mild (causes disruption but can be lived with) (2)
- Medium (difficult and persistent problem but you are learning to live with it/work around it) (3)
- Severe (persistent problem causing disruption to your normal life and your expectations) (4)

Skip To: Q40 If Does your puppy have an issue with chasing others or moving objects? = No problem at all (behaviour is absent or not worthy of note)

Please indicate who or what is being chased (please select all that apply)

- ☐ People (1)
- ☐ Other animals (2)
- ☐ Cars or vehicles (3)
- ☐ Other, please specify (4) ____

Page Break

Does your puppy have an issue with digging?

- No problem at all (behaviour is absent or not worthy of note) (1)
- Mild (causes disruption but can be lived with) (2)
- Medium (difficult and persistent problem but you are learning to live with it/work around it) (3)
- Severe (persistent problem causing disruption to your normal life and your expectations) (4)

Does your puppy have an issue with being alone?

- No problem at all (behaviour is absent or not worthy of note) (1)
- Mild (causes disruption but can be lived with) (2)
- Medium (difficult and persistent problem but you are learning to live with it/work around it) (3)
- Severe (persistent problem causing disruption to your normal life and your expectations) (4)

Does your puppy have an issue with noise fear?

- No problem at all (behaviour is absent or not worthy of note) (1)
- Mild (causes disruption but can be lived with) (2)
- Medium (difficult and persistent problem but you are learning to live with it/work around it) (3)
- Severe (persistent problem causing disruption to your normal life and your expectations) (4)

Page Break

Does your puppy have an issue with barking?

- No problem at all (behaviour is absent or not worthy of note) (1)
- Mild (causes disruption but can be lived with) (2)
- Medium (difficult and persistent problem but you are learning to live with it/work around it) (3)
- Severe (persistent problem causing disruption to your normal life and your expectations) (4)

Does your puppy have an issue with food stealing?

- No problem at all (behaviour is absent or not worthy of note) (1)
- Mild (causes disruption but can be lived with) (2)
- Medium (difficult and persistent problem but you are learning to live with it/work around it) (3)
- Severe (persistent problem causing disruption to your normal life and your expectations) (4)

Does your puppy have an issue with escaping?

- No problem at all (behaviour is absent or not worthy of note) (1)
- Mild (causes disruption but can be lived with) (2)
- Medium (difficult and persistent problem but you are learning to live with it/work around it) (3)
- Severe (persistent problem causing disruption to your normal life and your expectations) (4)

Page Break

Do you have an issue with your puppy being overactive?

- No problem at all (behaviour is absent or not worthy of note) (1)
- Mild (causes disruption but can be lived with) (2)
- Medium (difficult and persistent problem but you are learning to live with it/work around it) (3)
- Severe (persistent problem causing disruption to your normal life and your expectations) (4)

Does your puppy have an issue with growling, snapping or biting to protect something (like food, toys, bed etc)

- No problem at all (behaviour is absent or not worthy of note) (1)
- Mild (causes disruption but can be lived with) (2)
- Medium (difficult and persistent problem but you are learning to live with it/work around it) (3)
- Severe (persistent problem causing disruption to your normal life and your expectations) (4)

Does your puppy have an issue with separation related problems during the day?

- No problem at all (behaviour is absent or not worthy of note) (1)
- Mild (causes disruption but can be lived with) (2)
- Medium (difficult and persistent problem but you are learning to live with it/work around it) (3)
- Severe (persistent problem causing disruption to your normal life and your expectations) (4)

Page Break

Does your puppy appear to freeze up in fear or avoid specific things whilst outside?

- No problem at all (behaviour is absent or not worthy of note) (1)
- Mild (causes disruption but can be lived with) (2)
- Medium (difficult and persistent problem but you are learning to live with it/work around it) (3)
- Severe (persistent problem causing disruption to your normal life and your expectations) (4)

Does your puppy have an issue with disturbing you while you sleep at night?

- No problem at all (behaviour is absent or not worthy of note) (1)
- Mild (causes disruption but can be lived with) (2)
- Medium (difficult and persistent problem but you are learning to live with it/work around it) (3)
- Severe (persistent problem causing disruption to your normal life and your expectations) (4)

Does your puppy appear to have an issue with frustration, i.e resisting being restrained or struggling to cope when they cannot access something that they want?

- No problem at all (behaviour is absent or not worthy of note) (1)
- Mild (causes disruption but can be lived with) (2)
- Medium (difficult and persistent problem but you are learning to live with it/work around it) (3)
- Severe (persistent problem causing disruption to your normal life and your expectations) (4)

Page Break

Which of the following best describes your feelings about your puppy?

- ○ I feel very positive about sharing my life with my puppy (1)
- ○ Generally positive, but I have some concerns about sharing my life with my puppy (2)
- ○ I have substantial concerns about sharing my life with my puppy (3)
- ○ I regret getting a puppy (4)

When you think about the future with your puppy, which of the following best describes your feelings

- ○ I have the skills to cope with most challenges (1)
- ○ I can cope with most things, but am likely to still need advice in some areas (2)
- ○ I still need support to deal with quite a few aspects of life with my puppy (3)
- ○ I am very worried about my ability to cope with my puppy (4)

How well do you think your puppy is learning to fit in with your life i.e. life skills, play and training?

- ○ Very well (1)
- ○ Mostly fine, but there are some aspects he/she struggles with (2)
- ○ Not at all well (3)
- ○ He/she is totally out of control (4)
- ○ I don’t know (5)

Page Break

How well do you think you are equipped to deal with training your puppy as he gets older?

- Very well (1)
- Mostly fine, but there are some aspects he/she struggles with (2)
- Not at all well (3)
- I don’t know (4)

Is there any aspect of your puppy's behaviour that you are concerned about?

- No (1)
- Yes (2)

Skip To: Q74 If Is there any aspect of your puppy's behaviour that you are concerned about? = No

If you answered yes to the previous question, please explain why you're concerned

____

Page Break

Please select a point on the following scale that you feel best represents the quality of life your dog has with you, by clicking and dragging the slider to where you want it to be.
0 = I think he would rather be in a different home (NB you must click on the slider without moving it to register this choice)
100 = I think he has the best life he could wish for

	0 10 20 30 40 50 60 70 80 90 100
Click to write Choice 1 ()	

Please select a point on the following scale that you feel best represents how you feel about your life with your dog, by clicking and dragging the slider to where you want it to be.
0 = I am very seriously considering relinquishing him (NB you must click on the slider without moving it to register this choice)
100 = I could not be more satisfied

	0 10 20 30 40 50 60 70 80 90 100
Click to write Choice 1 ()	

End of Block: Block
