# Supplementary material for: Educational Apps and Dog Behavioural Problem Prevention: Associations Between the Zigzag Dog-Training App and Behavioural Problems
Source: Animals (Basel). 2025 Feb 12;15(4):520. doi: 10.3390/ani15040520 (PMC11851379; doi:10.3390/ani15040520)
Supplement: Supplementary file 1 [file animals-15-00520-s001.zip › animals-3398467-supplementary-proofreading.pdf]

Educational Apps and Dog Behaviour Problem Prevention: Associations Between the Zigzag Dog Training App and Behaviour Problems

Supplementary Material

Tom Rowland, Luciana de Assis, Carolyn Menteith, Lorna Winters, Helen Zulch and Daniel S Mills

Section 1: Zigzag app

Table S1. Summary of content from first four chapters of the Zigzag app.

**Chapter 1 - Basics**
Arriving home
Puppy Schedules - What you need to know
Toilet Training Introduction
Crate Training - Part 1
Sleep training
How to give a puppy the very best life possible - Marc Bekoff
A new home
Sit - Part 1
Continue toilet training
Crate Training - Part 2
Introduction to handling
Learning their name
Using a crate at night
Different surfaces
Recall - Part 1
Exploring surfaces
Playing Together
Crate fairy game
Toys for Chewing

Toys - Toilet Roll Tubes

Crate training - part 3

All about your Retriever

All about your Gun Dog

All about your Pastoral Dog

All about your Working Dog

All about your Terriers

All about your Sight Hounds

All about your Scent Hounds

All about your Toy Dogs

All about your Guardian Dogs

All about your Bull Dogs

Puppies need lots of sleep!

Toilet training at night time

Sights, sounds, and experiences

Why does my puppy cry at night?

Rewarding good behaviour with treats

Did you know... Teeth

Did you know... Labradors & Retrievers

Did you know..Gun Dogs

Did you know..Pastoral Dogs

Did you know..Working Dogs

Did you know..Terriers

Did you know..Sighthounds

Did you know..Scenthounds

Did you know..Toy Dogs

Did you know..Guardian Dogs

Did you know..Bull Breeds

Vaccination, fleas, and worming

Chapter 2 - Time to Play

Puppy Biting

Jumping up

Recall - Part 2

Meeting new people

The outside world

Sit - Part 2

Crate Training - Part 4

Car Journeys - Part 1

Recall - Part 3

Puppy Biting Checklist

Grooming - Part 1

Sit - Part 3

Retrieve - Part 1

Introduction to Bathing

Storm sounds

Being Alone - Part 1

Biting & Sleep

Keeping teeth off hands

Crate Training - Part 5

Exploring surfaces

When your pup has somehting they shouldnt have!

Learning and Having Fun

Remarkable Nose

Good Behaviour

Domestic Dogs

More about your breed - Retrievers

More about your breed - Gun Dogs

More about your breed - Pastoral Dogs

More about your breed - Working Dogs

More about your breed - Terriers

More about your breed - Sighthounds

More about your breed - Scenthounds

More about your breed - Toy Dogs

More about your breed - Guardian Dogs

More about your breed - Bull Breeds

Treating

Harness and Leads

Chapter 3 - Lifeskills

Play biting

Mouth, Eyes, Ears

Settle - Part 1

Biting - ReDirect to Toys

Sensory exploration inside

Introducing the harness

Firework sounds

Teething & Biting

Grooming - Part 2

Being alone - Part 2

Lead walking - Part 1

The vacuum cleaner

Meeting a stranger

Car journeys - part 2

Biting - Time Outs

Recall - Part 4

Toys - Muffin Tin

Down - Part 1

Traffic sounds

Retrieve - Part 2

Being Alone - Part 3

Crate Training - Part 6

The outside world

Bathing - Part 1

Sweating

Breed Behaviour - Retrievers

Breed Behaviour - Gun Dogs

Breed Behaviour - Pastoral Dogs

Breed Behaviour - Working Dogs

Breed Behaviour - Terriers

Breed Behaviour - Sighthounds

Breed Behaviour - Scenthounds

Breed Behaviour - Toy Dogs

Breed Behaviour - Guardian Dogs

Breed Behaviour - Bull Breeds

Fear Phases

Eyesight

Did you know...Retrievers

Did you know...Gun dogs

Did you know...Pastoral Dogs

Did you know...Working Dogs

Did you know...Terriers

Did you know...Sighthounds

Did you know...Scenthounds

Did you know...Toy Dogs

Did you know..Guardian Dogs

Did you know...Bull Breeds

Playing Together

Being Alone

Exercise Reminder

Chapter 4 - Bringing it all Together

Loose lead walking - Part 2

Recall - Game

Down - Part 2

Follow me - Part 1

Recall - Part 5

Being alone - Part 4

Loose lead - Part 3

Sit - Part 4

Bathing - Part 2

Settle - Part 2

Tails

Vacuum cleaner sounds

Retrieving toys

Lead walking - Part 4

Car Journeys - Part 3

Down - Part 3

Meeting another dog

Recall - Part 6

Toys - Bottle Dispenser

Sit - Part 5

Leave It - Intro

Sensitive Hearing

About Dogs

Wet Nose

Harness over collar

Sleeping

Training opportunities

Kongs

Exercise Reminder

Section 2: Breed of dogs

Table S2. Frequency of breeds in the Zigzag and no training groups.

Breed	No training	Zigzag	Total
Labrador Retriever	16	20	36
Cocker Spaniel	17	12	29
Cockapoo	15	10	25
Na's	5	17	22
Mixed Breed	10	5	15

Border Collie	7	6	13
Cavapoo	6	3	9
German Shepherd Dog	2	7	9
Golden Retriever	4	5	9
Siberian Husky	5	4	9
Dachshund Miniature Smooth Haired	4	4	8
English Springer Spaniel	3	5	8
Other	4	4	8
French Bulldog	6	1	7
Labradoodle	3	4	7
Shih Tzu	4	3	7
Staffordshire Bull Terrier	5	2	7
Crossbreed	2	4	6
Whippet	2	4	6
Goldendoodle	3	2	5
Miniature Schnauzer	2	3	5
West Highland White Terrier	3	2	5
Beagle	3	1	4
Chihuahua	2	2	4
Yorkshire Terrier	4	0	4
Fox Terrier Wire	0	4	4
American Pit Bull Terrier	3	0	3
Cavapoochon	1	2	3
Chow Chow	2	1	3
Maltipoo	3	0	3
Rottweiler	3	0	3
American Bulldog	2	0	2
Australian Cattle Dog	0	2	2
Australian Shepherd	1	1	2
Bichon Frise	2	0	2
Border Terrier	0	2	2
Cavalier King Charles Spaniel	2	0	2
Dachshund Miniature Long Haired	2	0	2
Dalmatian	2	0	2
English Cocker Spaniel	1	1	2
Pomeranian	2	0	2
Poodle Standard	1	1	2
Poodle Toy	2	0	2
Schnauzer	2	0	2

Shetland Sheepdog	2	0	2
Sprocker	1	1	2
Weimaraner	2	0	2
Hungarian Vizsla	0	2	2
Jack Russell Terrier	0	2	2
Large Mixed Breed	0	2	2
Affenpinscher	1	0	1
Airedale Terrier	1	0	1
Akita	1	0	1
American Cocker Spaniel	0	1	1
Australian Kelpie	1	0	1
Australian Terrier	0	1	1
Barbet	1	0	1
Beauceron	0	1	1
Bedlington Terrier	1	0	1
Belgian Shepherd Dog Groenendael	1	0	1
Belgian Shepherd Dog Malinois	0	1	1
Bernadoodle	0	1	1
Boxer	0	1	1
Brittany	1	0	1
Bull Terrier	1	0	1
Cairn Terrier	1	0	1
Cavachon	0	1	1
Chihuahua Long Coat	0	1	1
Chihuahua Smooth Coat	1	0	1
Cockalier	0	1	1
Collie Rough	0	1	1
Dachshund Long Haired	1	0	1
Dutch Shepherd	1	0	1
Gerberian Shepsky	1	0	1
Hungarian Wire Haired Vizsla	1	0	1
Irish Setter	1	0	1
Japanese Spitz	1	0	1
Lurcher	1	0	1
Parson Russell Terrier	1	0	1
Schnoode	1	0	1
Shorkie	1	0	1
English Setter	0	1	1
German Shorthaired Pointer	0	1	1

Italian Greyhound	0	1	1
Keeshond	0	1	1
Lakeland Terrier	0	1	1
Maremma Sheepdog	0	1	1
Papillon	0	1	1
Pomapoo	0	1	1
Poodle Miniature	0	1	1
Pug	0	1	1
Tibetan Terrier	0	1	1
Welsh Terrier	0	1	1
**Total**	194	173	367

Section 3: Model results

Please note that in all the following tables the predictors 1|2, 2|3, and 3|4 refer to the threshold parameters of the ordinal regressions. Further, age, sex, where from and health predictors all refer to characteristics of the dog and not their owners.

Table S3. Full ordinal logistic regression model table for chewing.

	**Chewing**		
Predictors	*Log-Odds*	*CI*	*p*
1|2	-2.13	-2.69 – -1.58	**<0.001**
2|3	0.21	-0.28 – 0.71	0.392
3|4	2.36	1.68 – 3.04	**<0.001**
completion	-1.16	-1.82 – -0.50	**0.001**
age	-0.11	-0.16 – -0.06	**<0.001**
sex [male]	0.15	-0.28 – 0.58	0.489
where from [1]	0.98	0.23 – 1.73	**0.010**
health [Yes, please	-0.93	-1.84 – -0.02	**0.044**
Observations	313		

R^2 Nagelkerke	0.144

Table S4. Full ordinal logistic regression model table for play.biting.

	**Play.biting**		
Predictors	*Log-Odds*	*CI*	*p*
1|2	-2.16	-2.71 – -1.61	**<0.001**
2|3	-0.20	-0.69 – 0.28	0.414
3|4	1.71	1.12 – 2.30	**<0.001**
completion	-0.38	-1.02 – 0.26	0.241
age	-0.15	-0.20 – -0.10	**<0.001**
sex [male]	0.21	-0.22 – 0.63	0.338
where from [1]	0.41	-0.30 – 1.13	0.259
health [Yes, please specify]	-0.23	-1.10 – 0.65	0.614
Observations	313		
R^2 Nagelkerke	0.155		

Table S5. Full ordinal logistic regression model table for house.soiling.

	**House.soiling**		
Predictors	*Log-Odds*	*CI*	*p*
1|2	-1.86	-2.42 – -1.30	**<0.001**
2|3	0.07	-0.45 – 0.59	0.792
3|4	1.68	1.02 – 2.35	**<0.001**
completion	-1.14	-1.82 – -0.46	**0.001**
age	-0.17	-0.23 – -0.11	**<0.001**
sex [male]	-0.37	-0.81 – 0.07	0.097
where from [1]	0.39	-0.38 – 1.15	0.319
health [Yes, please	0.10	-0.81 – 1.00	0.831

Observations	313		
R^2 Nagelkerke	0.183		

Table S6. Full ordinal logistic regression model table for familiar.agg.

	**Familiar.agg**		
Predictors	*Log-Odds*	*CI*	*p*
1|2	1.74	0.96 – 2.51	**<0.001**
2|3	3.04	2.15 – 3.93	**<0.001**
3|4	5.81	3.72 – 7.90	**<0.001**
completion	-1.50	-2.69 – -0.31	**0.014**
age	-0.03	-0.10 – 0.04	0.343
sex [male]	0.80	0.11 – 1.49	**0.024**
where from [1]	0.66	-0.34 – 1.66	0.194
health [Yes, please	-1.50	-3.56 – 0.55	0.151
Observations	313		
R^2 Nagelkerke	0.081		

Table S7. Full ordinal logistic regression model table for unfamiliar.agg.

	**Unfamiliar.agg**		
Predictors	*Log-Odds*	*CI*	*p*
1|2	2.96	2.01 – 3.92	**<0.001**
2|3	5.09	3.82 – 6.35	**<0.001**
3|4	6.72	4.56 – 8.88	**<0.001**
completion	-0.35	-1.50 – 0.80	0.554
age	0.03	-0.04 – 0.10	0.436
sex [male]	0.97	0.16 – 1.78	**0.019**

where from [1]	1.30	0.32 – 2.27	**0.009**
health [Yes, please	0.16	-1.16 – 1.48	0.811
Observations	312		
R^2 Nagelkerke	0.072		

Table S8. Full ordinal logistic regression model table for conspecific.agg.

	**Conspecific.agg**		
Predictors	*Log-Odds*	*CI*	*p*
1|2	2.12	1.35 – 2.89	**<0.001**
2|3	3.96	2.96 – 4.95	**<0.001**
3|4	5.52	3.94 – 7.09	**<0.001**
completion	-1.17	-2.42 – 0.07	0.064
age	0.05	-0.01 – 0.12	0.092
sex [male]	-0.03	-0.70 – 0.63	0.919
where from [1]	1.76	0.90 – 2.62	**<0.001**
health [Yes, please	-1.40	-3.48 – 0.67	0.185
Observations	311		
R^2 Nagelkerke	0.121		

Table S9. Full ordinal logistic regression model table for jumping.

	**Jumping**		
Predictors	*Log-Odds*	*CI*	*p*
1|2	-1.47	-1.99 – -0.95	**<0.001**
2|3	0.68	0.18 – 1.18	**0.007**
3|4	2.62	1.99 – 3.25	**<0.001**
completion	-0.34	-0.96 – 0.28	0.288

age	0.03	-0.01 – 0.08	0.143
sex [male]	-0.46	-0.88 – -0.03	**0.035**
where from [1]	0.26	-0.48 – 0.99	0.492
health [Yes, please	-0.03	-0.92 – 0.86	0.953
Observations	311		
R^2 Nagelkerke	0.047		

Table S10. Full ordinal logistic regression model table for car travel.

	**car.travel**		
Predictors	*Log-Odds*	*CI*	*p*
1|2	0.77	0.23 – 1.31	**0.005**
2|3	2.59	1.95 – 3.24	**<0.001**
3|4	3.70	2.85 – 4.55	**<0.001**
completion	-0.06	-0.77 – 0.66	0.876
age	0.01	-0.04 – 0.06	0.795
sex [male]	0.03	-0.45 – 0.50	0.912
where from [1]	0.17	-0.64 – 0.97	0.685
health [Yes, please	0.11	-0.85 – 1.06	0.827
Observations	311		
R^2 Nagelkerke	0.008		

Table S11. Full ordinal logistic regression model table for chasing.

chasing			
Predictors	*Log-Odds*	*CI*	*p*
1|2	0.56	0.06 – 1.06	**0.027**
2|3	2.32	1.74 – 2.90	**<0.001**
3|4	4.26	3.32 – 5.19	**<0.001**
completion	0.18	-0.48 – 0.83	0.593
age	0.05	-0.00 – 0.09	0.051
sex [male]	-0.03	-0.48 – 0.41	0.879
where from [1]	0.06	-0.72 – 0.83	0.887
health [Yes, please	-0.42	-1.37 – 0.53	0.383
Observations	311		
R^2 Nagelkerke	0.029		

Table S12. Full ordinal logistic regression model table for digging.

Digging			
Predictors	*Log-Odds*	*CI*	*p*
1|2	0.49	-0.02 – 1.01	0.062
2|3	2.26	1.67 – 2.85	**<0.001**
3|4	4.07	3.17 – 4.96	**<0.001**
completion	0.18	-0.48 – 0.85	0.594
age	-0.00	-0.05 – 0.05	0.988
sex [male]	0.35	-0.10 – 0.80	0.125
where from [1]	0.48	-0.27 – 1.23	0.208
health [Yes, please	-0.12	-1.00 – 0.76	0.787
Observations	309		

R^2 Nagelkerke	0.040

Table S13. Full ordinal logistic regression model table for being alone.

	**being.alone**		
Predictors	*Log-Odds*	*CI*	*p*
1|2	-0.85	-1.35 – -0.35	**0.001**
2|3	0.59	0.10 – 1.09	**0.019**
3|4	2.08	1.48 – 2.68	**<0.001**
completion	-0.39	-1.05 – 0.26	0.237
age	-0.07	-0.11 – -0.02	**0.007**
sex [male]	-0.12	-0.54 – 0.30	0.569
where from [1]	0.65	-0.08 – 1.37	0.081
health [Yes, please	0.63	-0.17 – 1.43	0.123
Observations	309		
R^2 Nagelkerke	0.061		

Table S14. Full ordinal logistic regression model table for noise fear.

	**noise.fear**		
Predictors	*Log-Odds*	*CI*	*p*
1|2	0.68	0.14 – 1.22	**0.013**
2|3	2.92	2.24 – 3.60	**<0.001**
3|4	4.62	3.51 – 5.73	**<0.001**
completion	-0.78	-1.53 – -0.03	**0.042**
age	0.00	-0.05 – 0.05	0.958
sex [male]	0.38	-0.09 – 0.85	0.116
where from [1]	0.53	-0.25 – 1.31	0.182

health [Yes, please	1.13	0.22 – 2.05	**0.015**
Observations	309		
R^2 Nagelkerke	0.084		

Table S15. Full ordinal logistic regression model table for barking.

	**barking**		
Predictors	*Log-Odds*	*CI*	*p*
1|2	0.06	-0.44 – 0.55	0.827
2|3	1.96	1.41 – 2.52	**<0.001**
3|4	3.84	3.00 – 4.68	**<0.001**
completion	-0.73	-1.40 – -0.06	**0.032**
age	0.03	-0.01 – 0.08	0.151
sex [male]	0.06	-0.38 – 0.49	0.795
where from [1]	0.80	0.07 – 1.52	**0.031**
health [Yes, please	-0.12	-1.02 – 0.78	0.797
Observations	309		
R^2 Nagelkerke	0.059		

Table S16. Full ordinal logistic regression model table for food stealing.

	**food.stealing**		
Predictors	*Log-Odds*	*CI*	*p*
1|2	0.30	-0.22 – 0.82	0.260
2|3	1.69	1.12 – 2.26	**<0.001**
3|4	3.18	2.41 – 3.96	**<0.001**
completion	-0.35	-1.05 – 0.36	0.335
age	-0.01	-0.05 – 0.04	0.785

sex [male]	-0.13	-0.59 – 0.32	0.569
where from [1]	-0.17	-0.96 – 0.62	0.674
health [Yes, please	-0.03	-0.98 – 0.93	0.956
Observations	309		
R^2 Nagelkerke	0.012		

Table S17. Full ordinal logistic regression model table for escaping.

	**escaping**		
Predictors	*Log-Odds*	*CI*	*p*
1|2	1.09	0.42 – 1.76	**0.001**
2|3	2.93	2.06 – 3.80	**<0.001**
3|4	4.16	2.88 – 5.45	**<0.001**
completion	-1.72	-2.95 – -0.50	**0.006**
age	-0.02	-0.08 – 0.05	0.607
sex [male]	-0.22	-0.83 – 0.39	0.479
where from [1]	0.75	-0.13 – 1.64	0.096
health [Yes, please	-0.74	-2.25 – 0.77	0.336
Observations	309		
R^2 Nagelkerke	0.084		

Table S18. Full ordinal logistic regression model table for overactive.

	**overactive**		
Predictors	*Log-Odds*	*CI*	*p*
1|2	-0.54	-1.04 – -0.04	**0.036**
2|3	1.53	0.98 – 2.07	**<0.001**
3|4	3.45	2.58 – 4.31	**<0.001**

completion	-0.11	-0.75 – 0.54	0.746
age	-0.05	-0.10 – -0.01	**0.024**
sex [male]	0.01	-0.42 – 0.44	0.954
where from [1]	0.27	-0.47 – 1.00	0.477
health [Yes, please	0.06	-0.84 – 0.95	0.902
Observations	309		
R^2 Nagelkerke	0.033		

Table S19. Full ordinal logistic regression model table for resource.agg.

	**resource.agg**		
Predictors	*Log-Odds*	*CI*	*p*
1|2	1.79	1.04 – 2.54	**<0.001**
2|3	3.35	2.44 – 4.26	**<0.001**
3|4	4.68	3.35 – 6.00	**<0.001**
completion	-1.11	-2.31 – 0.09	0.069
age	0.01	-0.06 – 0.07	0.795
sex [male]	0.07	-0.59 – 0.72	0.840
where from [1]	0.84	-0.09 – 1.77	0.078
health [Yes, please	0.53	-0.55 – 1.60	0.334
Observations	309		
R^2 Nagelkerke	0.045		

Table S20. Full ordinal logistic regression model table for separation.during.day.

	**separation.during.day**		
Predictors	*Log-Odds*	*CI*	*p*
1|2	-0.43	-0.94 – 0.07	0.094
2|3	1.20	0.67 – 1.73	**<0.001**
3|4	2.33	1.70 – 2.97	**<0.001**
completion	-0.37	-1.04 – 0.30	0.276
age	-0.04	-0.09 – 0.01	0.107
sex [male]	-0.16	-0.59 – 0.27	0.458
where from [1]	0.27	-0.46 – 1.01	0.462
health [Yes, please	0.04	-0.80 – 0.88	0.929
Observations	309		
R^2 Nagelkerke	0.026		

Table S21. Full ordinal logistic regression model table for fear outside.

	**fear.outside**		
Predictors	*Log-Odds*	*CI*	*p*
1|2	0.89	0.30 – 1.48	**0.003**
2|3	2.60	1.90 – 3.30	**<0.001**
3|4	4.06	3.02 – 5.10	**<0.001**
completion	-0.53	-1.34 – 0.29	0.208
age	-0.03	-0.08 – 0.03	0.311
sex [male]	0.18	-0.34 – 0.70	0.499
where from [1]	0.40	-0.44 – 1.23	0.353
health [Yes, please	1.07	0.14 – 2.01	**0.025**
Observations	305		

R^2 Nagelkerke	0.040

Table S22. Full ordinal logistic regression model table for sleep disturbance.

	**sleep.disturbance**		
Predictors	*Log-Odds*	*CI*	*p*
1|2	0.79	0.18 – 1.39	**0.011**
2|3	3.14	2.31 – 3.98	**<0.001**
3|4	4.38	3.12 – 5.64	**<0.001**
completion	-0.25	-1.07 – 0.57	0.546
age	-0.04	-0.10 – 0.02	0.177
sex [male]	-0.07	-0.60 – 0.46	0.797
where from [1]	-0.02	-0.93 – 0.88	0.957
health [Yes, please	1.13	0.19 – 2.07	**0.019**
Observations	305		
R^2 Nagelkerke	0.046		

Table S23. Full ordinal logistic regression model table for frustration.

	**frustration**		
Predictors	*Log-Odds*	*CI*	*p*
1|2	0.01	-0.51 – 0.52	0.977
2|3	1.74	1.17 – 2.31	**<0.001**
3|4	3.51	2.63 – 4.38	**<0.001**
completion	-0.01	-0.69 – 0.66	0.965
age	-0.05	-0.10 – 0.00	0.056
sex [male]	-0.00	-0.45 – 0.45	0.984
where from [1]	0.40	-0.34 – 1.13	0.290

health [Yes, please	0.32	-0.56 – 1.19	0.480
Observations	305		
R^2 Nagelkerke	0.028		
